# Digital Inclusion in a Scottish National Pulmonary Hypertension Population

**DOI:** 10.1002/pul2.70135

**Published:** 2025-07-27

**Authors:** Jamie Ingram, Harrison Stubbs, Stephanie Lua, Melanie Brewis, Martin Johnson, Colin Church

**Affiliations:** ^1^ Scottish Pulmonary Vascular Unit Golden Jubilee National Hospital Glasgow UK; ^2^ School of Cancer Sciences University of Glasgow Glasgow UK; ^3^ School of Cardiovascular and Metabolic Health University of Glasgow Glasgow UK

**Keywords:** digital health technology, digital inclusion, health inequality, telemedicine, wearable devices

## Abstract

To evaluate current digital inclusion in the Scottish pulmonary hypertension population, a paper questionnaire was offered to the entirety of patients with pulmonary arterial hypertension in Scotland. The Scottish Index of Multiple Deprivation was used to stratify patients into deprivation deciles. 464 patients returned questionnaires (86%). 91% had reliable internet access. 89% had access to an internet‐enabled device. 71% used the internet daily. The most common barriers to increased internet usage were confidence with technology (19%) and lack of perceived personal benefit (7%). 54% would like virtual healthcare to complement in person review and 58% would like to monitor their health digitally. Older patients were less likely to use the internet and had less desire for virtual healthcare. Rural living did not negatively impact access to the internet. Younger, more rural, and less deprived patients currently use and desire more online exercise. Deprived patients were less likely to have internet access or internet enabled devices, more likely to have no device or a mobile without internet, and had less desire for virtual healthcare or digital health monitoring. Most patients have the means of accessing the internet and support virtual healthcare in addition to direct clinician contact. However, digital engagement was lower in older and more deprived patients. The high response rate supports paper over online survey methodology for future digital inclusion research. Future digital healthcare strategies need to integrate this knowledge to minimize age‐ and deprivation‐related inequity.

As digital healthcare becomes more integrated in the management of patients with pulmonary vascular disease, there is a corresponding requirement for increased digital access and engagement amongst the patient population to avoid further increases in healthcare inequity. Technological advances have yielded wearable and implantable devices for remote health monitoring, smartphone health and fitness apps, and virtual consultations. These developments can improve patient autonomy, wellbeing and convenience, however, are not equally accessible. Digital inclusion refers to the equal opportunity for individuals to engage in the digital world irrespective of their circumstances and requires not only access to digital devices and the internet, but also effective digital skills, support and confidence to utilize them. Although 96% of British households had internet access in February 2020, only 80% of households with an adult aged 65 or over did [1]. These statistics may overestimate access with 80% of respondents completing the survey online, highlighting an inherent limitation to online survey methodology; digital access and skills are required to complete them.

Patients with pulmonary hypertension (PH), a chronic disease of the pulmonary vasculature, are increasingly exposed to technology. App‐based 6‐min walk tests (6MWT—a routine assessment of exercise capacity) [2] implantable pulmonary artery pressure monitors [3] and mobile health interventions [4] are a focus in ongoing PH research and echo trends seen in the general cardiorespiratory field [5, 6]. However, the pulmonary hypertension patient perspective on the use of technology in healthcare has not been widely reported. A Pulmonary Hypertension Association UK (PHA UK) survey provides perhaps the best account of this, comparing the ownership and use of digital devices and social media alongside patient views on health monitoring using digital devices [7], albeit also limited by online survey methodology.

Pulmonary hypertension is a rare disease, and as such all pulmonary hypertension patients in Scotland are cared for by a single, nationally designated center, the Scottish Pulmonary Vascular Unit (SPVU), based in Glasgow. Patients often travel significant distances for face‐to‐face review and require regular follow up. These conditions may benefit from the inclusion of telemedicine approaches; however, these must be implemented in a manner that does not further exacerbate health inequity. Remote health monitoring may aid the early detection of deterioration allowing prompt review and optimization by the specialist service. We sought to develop a better understanding of how patients with PH in Scotland interact with digital technologies and assess their views on future engagement, with a particular interest in detecting areas susceptible to inequity such as age, deprivation and geographical remoteness.

## 1 Methods

A paper questionnaire covering several areas related to digital inclusion was created and offered to all prevalent patients with precapillary pulmonary hypertension, belonging to WHO groups 1, 4, and 5, under the care of the SPVU at outpatient clinic review. The questionnaire was initially piloted with a focus group of 8 patients to ensure it was easy to understand, complete and achieved face validity. In March 2023 a list was generated from clinical records of all patients attending the SPVU outpatient clinic with precapillary PH. Questionnaire data were collected over a 12‐month period from March 2023 to March 2024. Any patients who were not seen face‐to‐face or appointed to a clinic within that time frame were sent the questionnaire by post in February 2024.

The survey included a covering sheet explaining the rationale for the questionnaire, an explanation of what digital healthcare means, and the tests or technologies included within the scope of digital healthcare. Thirteen questions were included, each with checkboxes to tick the answer most appropriate to the patient. Patients were given the option to include their postcode if they wished to help identify geographical areas where access to digital technology was poor and include their age bracket (e.g., 18–30 years) to aid analysis of age‐related inequity. A free text box was provided for patients to note additional comments. The questionnaire was provided in English only, however, would have been translated if required.

Questionnaire results were transferred to a Microsoft Excel spreadsheet on an NHS data encrypted, password protected computer. The Scottish Index of Multiple Deprivation (SIMD) was used to convert postcodes to SIMD data zones to allow deprivation‐ and location‐based analysis. Consultation with the West of Scotland Research Ethics Service determined that the study did not require ethical review.

## 2 Results

### 2.1 Questionnaire Responses

**Figure 1:**
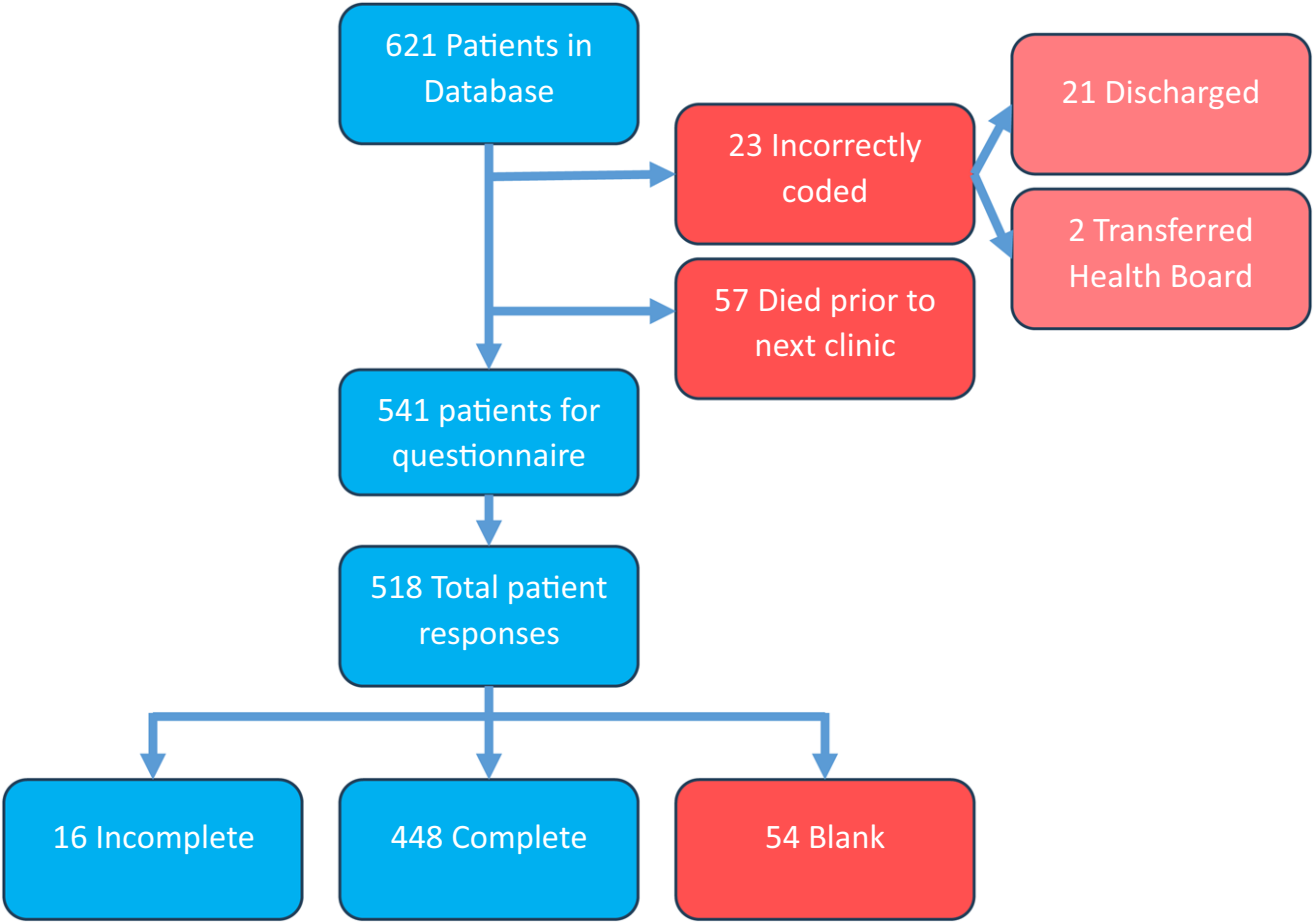
Data collection flowchart.

A list of 621 prevalent PH patients under the care of the SPVU was retrieved from clinical records in March 2023. After removing data for patients who had died or were discharged before clinic review this reduced to 541 patients who were offered questionnaires. The majority were offered questionnaires at clinic, but 91 (16·8%) were mailed to patients who were not seen in clinic within the study period. 518 questionnaires were returned, of which 448 were complete, 16 were incomplete, and 54 were blank. Data from 464 patients, or 85·8% of the SPVU patient population were available for analysis (Figure 1).

### 2.2 Patient Demographics

Patients were divided into 5 age groups on the questionnaire, with age documented by 442 respondents (Table 1). Most patients were in the 51–64 or 65–80 years age bracket. Deprivation data were analysed using the SIMD to convert the 414 postcodes provided by respondents to SIMD numbers. Subsequently patients were arranged into their overall SIMD decile, ranking from 1 to 10 with 1 representing the most deprived and 10 being least deprived, as well as into their urban/rural classification. There was an equal distribution noted between the deprivation deciles and most patients were located in urban areas.

**Table 1:** Patient demographics.

Patient demographics								
Age	Patient number	%	SIMD decile	Patient number	%	Urban‐Rural class	Patient number	%
18–30	21	4.8	1	40	9.7	Large Urban	132	31.9
31–50	74	16.7	2	52	12.6	Other Urban	152	36.7
51–64	141	31.9	3	43	10.4	Accessible Small Town	43	10.4
65–80	182	41.2	4	40	9.7	Remote Small Town	10	2.4
> 81	23	5.2	5	45	10.9	Accessible Rural	58	14.0
			6	45	10.9	Remote Rural	19	4.6
			7	35	8.5			
			8	45	10.9	Accessible	385	93.0
			9	39	9.4	Remote	29	7.0
			10	30	7.2			

### 2.3 Questionnaire Results

#### 2.3.1 Internet‐Related Questions

Patients were asked if they had access to internet that was “consistent and reliable” as this was felt to be important in allowing reasonable internet use. The main findings are as follows:

- The majority of patients (91.5%) have good internet access, with most declaring home broadband as their internet supply (87.3%) and others using their mobile phone internet (59.8%).
- More than half of patients (56%) have two or more internet‐enabled devices.
- 8.5% of patients declare no reliable access.

Most patients have at least one device from which they can access the internet (89.3%) with the most commonly owned device being a smartphone (79.5%). 47.5% of patients have tablets and similar numbers were seen for laptops/desktop computers (46.4%). 14.7% own a smartwatch. Significant numbers of patients (10.7%) have no internet‐enabled devices, and a few (3.6%) had no devices whatsoever.

Patients use the internet regularly with more than 80% of patients using the internet at least on a weekly basis and 70·9% using it every day. However, over 1 in 10 claims to never use the internet. While most patients have personal email addresses (85.1%), 1 in 7 does not.

Previous reports have suggested that there exists an age‐related inequity when considering digital access [1], therefore questions targeting potential barriers to internet use that may affect this demographic were included in the questionnaire. Patients were asked whether the internet “scared“ them with 15.9% agreeing that it did. The most common barrier within our population was low confidence in using technology (18.8% of patients) and over a third of patients rely on others for online tasks. There were also clear health barriers which included eyesight, hearing loss and finger dexterity affecting around 1 in 14. Less common barriers were financial expense, lack of devices, poor internet signal and feeling the internet was not worthwhile using. The majority of patients, however, felt there were no barriers to internet use (61.9%) (Table 2).

**Table 2:** Internet‐related study questions.

Study question	Number of respondents	No answer	Patients answering “Yes”	
			Absolute number	% of patients
**Internet access (total)**	448	**0**	410	91.5%
Home broadband			391	87.3%
Mobile phone internet			268	59.8%
“Other”/Unspecified			7	1.6%
Two or more internet sources			251	56.0%
**No internet access**			38	8.5%
**Digital device access (total)**	448	0	432	96.4%
Mobile phone with internet			356	79.5%
Tablet/iPad			213	47.5%
Laptop/Desktop computer			208	46.4%
Smartwatch			66	14.7%
Mobile with no internet			46	10.3%
No internet‐enabled device			48	10.7%
No device of any kind			16	3.6%
**Email address**	437	11	372	85.1%
**Frequency of internet use**	447	1		
Daily			317	70.9%
Weekly			44	9.8%
Monthly			8	1.8%
Rarely			27	6.0%
Never			51	11.4%
**Does the internet scare patients**	446	2	71	15.9%
**Do patients rely on others for online tasks**	446	2	164	36.8%
**Patient barriers to internet use**	446	2		
Confidence with technology			84	18.8%
Health barriers			31	7.0%
Internet is “not worthwhile”			26	5.8%
Lack of devices			25	5.6%
Poor internet signal			22	4.9%
Expensive			9	**2**.**0%**
No barriers to internet use			276	61.9%

### 2.4 Virtual Healthcare and Digital Monitoring Questions

Virtual healthcare, a subset of telemedicine which refers to patients and clinicians meeting remotely with the help of mobile apps and videoconferencing, has become more popular in the wake of the COVID‐19 pandemic [8]. Patients were asked if they would like virtual or online methods of healthcare to complement, but not replace, their face‐to‐face appointments. Over half of patients (54.4%) would be in favor of this approach.

A question on the monitoring of health digitally was also included as a plethora of health and fitness smartphone apps and functions are now available to track your wellbeing. Again, more than half (57.6%) would like to use digital health monitoring (Table 3).

**Table 3:** Virtual healthcare and digital monitoring questions.

Study question	Number of Respondents	No answer	Patients answering “Yes”	
			Absolute number	% of patients
Would you like virtual methods of healthcare alongside face‐to‐face appointments?	441	7	240	54.4%
Would you like to monitor your health digitally?	441	7	254	57.6%

### 2.5 Online Exercise Questions

Exercise is now considered beneficial for patients with pulmonary hypertension as reflected in the 2022 ESC/ERS guidelines [9], however in‐person supervised exercise programs are often faced with poor attendance and non‐completion [10]. Online methods of directed exercise could overcome barriers such as distance and cost of travel.

Patients were asked if they used any online resources to help them exercise or stay healthy such as YouTube videos, online gym classes or home exercise bike applications such as Peloton. Only 1 in 10 patients currently use some form of exercise online, predominantly YouTube content, however 63% of patients expressed they would like access to any future online PH‐tailored exercise resources. More specifically, 32·4% of patients would use group PH exercise classes and 41.3% would use online exercise video content, with 10.7% of patients wishing to use both (Table 4).

**Table 4:** Online exercise questions.

Study question	Number of Respondents	No answer	Patients answering “Yes”	
			Absolute number	% of patients
Do you use any online exercise resources?	441	7	47	10·7%
Would you like access to future online exercise resources?	441	7		
Total (at least one resource)			278	63·0%
Group classes			143	32·4%
Exercise videos			182	41·3%
Both			47	10·7%

### 2.6 Analysis of Age and Digital Inclusion Parameters

**Figure 2:**
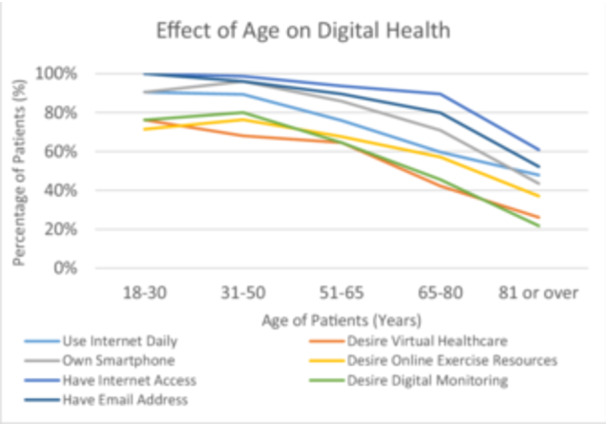
Effect of age on digital health.

Increasing age is associated with reduction in the means of digital access and less desire to engage digitally (see Figure 2). Older patients use the internet less frequently and 43% of those aged over 80 years claimed to never go online. The likelihood of owning most internet‐enabled devices consistently drops with age. However, tablets were owned more often by older‐aged than middle‐aged patients and were more common (57%) than smartphones (43%) in those over 80 years.

No patients aged over 80 currently use online exercise resources and only 37% would consider it. In contrast, around 70% of those up to the age of 65 would try some form of online exercise resource. Exercise videos were most popular amongst the 31–50‐year age group whereas group exercise was valued more by the youngest patients.

Analysis of the perceived barriers to internet use also revealed age‐related trends. Older patients generally find the internet scary, rely on others for help, lack confidence in technology, have healthcare barriers and have no internet devices, whereas younger groups identified poor signal and expense as the major problems. Older patients also had less interest in virtual healthcare and digital monitoring.

### 2.7 Analysis of Deprivation Status and Digital Inclusion Parameters

**Figure 3:**
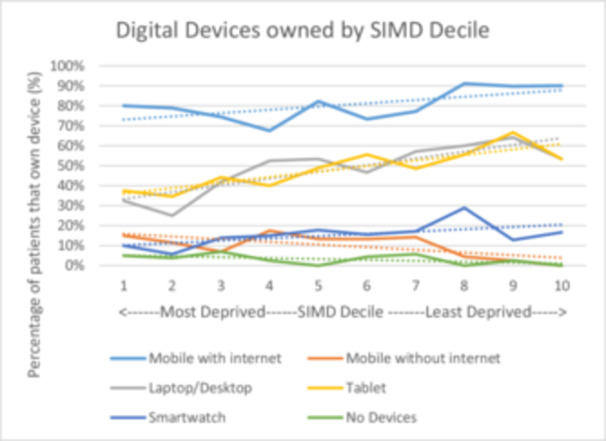
Digital devices owned by SIMD decile. Percentages of patients owning each device plotted against their SIMD decile with best fit lines included.

The degree of deprivation experienced by a patient influences their digital access. Trends suggest that as deprivation increases, ownership of internet‐enabled digital devices falls. This was evident for internet‐enabled mobile phones, laptops/desktops, smartwatches and tablets. More deprived patients were more likely to have phones without internet or to have no devices (See Figure 3).

**Figure 4:**
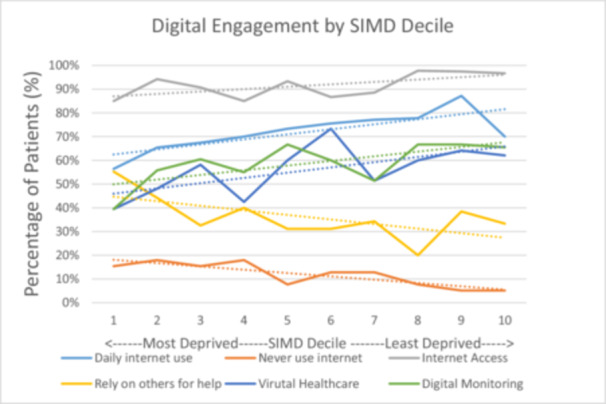
Digital engagement by SIMD decile. Percentage of patients plotted against SIMD decile for each digital health parameter with best fit lines included.

Less deprived patients were more likely to have internet access, use the internet daily and value virtual healthcare and digital monitoring. More deprived patients more frequently stated they never used the internet and relied on others for help with online tasks (Figure 4).

### 2.8 Analysis of Urban‐Rural Classification and Digital Inclusion Parameters

The Scottish Government Urban Rural Classification 2020 [11] provides a way to define remote and rural areas allowing comparison of digital inclusion between accessible and more remote areas of Scotland. Accessible areas are defined as those within a 30‐min drive of a settlement with a population of 10,000 or more, otherwise they are considered to be remote.

**Figure 5:**
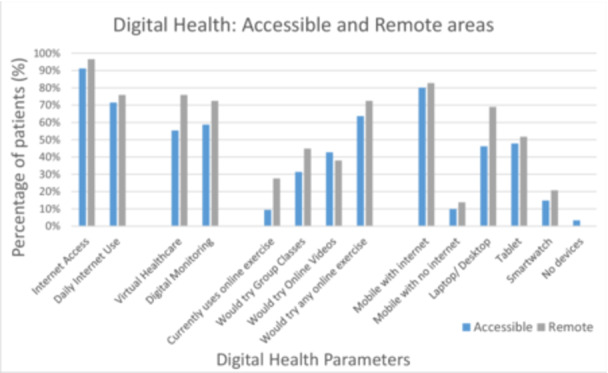
Digital health parameters and accessible vs remote living.

Internet access, daily internet use and ownership of all types of digital devices was more common in patients living in remote areas. Patients living rurally were more interested in virtual healthcare consultations alongside ways to monitor their health digitally. With regard to online exercise, those living in remote areas were substantially more likely than those in accessible areas to currently be using online exercise resources (28% vs. 9%). They also had more desire for online group exercise classes and a higher overall interest in PH‐specific online exercise (See Figure 5).

## 3 Discussion

This study provides greater insight into the digital access and engagement of a national patient group, with responses from 85.8% of the pulmonary hypertension population in Scotland. This high response rate supports the paper‐based, in‐person methodology used which has provided a representative overview of patients from all age groups, social backgrounds and regions of Scotland and is a strength of this study. Many surveys previously exploring digital access for patients have used online methods to collect data which can clearly lead to selection bias. Whether this study's results are generalizable to other patient groups with another disease remains to be determined and requires further work.

Reliable access to the internet was claimed by 91.5% of the study population, which, while lower than the 96% of households in Great Britain in 2020 [1], may reflect relatively poorer internet access in Scotland where access was at a record high of 91% in 2022 [12]. Nationally, these statistics have continuously improved over recent years. However, internet access is only one of a multitude of complex factors needed to address the digital divide; simply being “online” isn't enough. The Digital Poverty Alliance suggests five determinants of digital poverty: (a) devices and connectivity, (b) access, (c) capability, (d) motivation, and (e) support [13]. This means that patients need not only to possess the means to go online, but also the desire and digital skills to use it safely with user‐friendly resources, and adequate technical support on hand as required. These areas are important to consider as healthcare professionals striving to enhance patient care through the use of digital technologies and any new technology‐based interventions should incorporate the means to maximize the accessibility to all patients, particularly those in typically underserved populations.

In older adults, “technophobia,” lower digital literacy [14] and health‐related barriers are among factors recognized to contribute to low digital engagement. These are supported by results from our study group since the majority of those identifying the internet as “scary” and citing low confidence with technology as a barrier to internet use were from older age groups. Methods to identify those with adequate digital literacy have been developed, such as the eHealth Literacy Assessment Toolkit [15]. Such approaches could help to screen for patients that may require enhanced support to ensure exclusion is minimized, while providing confidence that others will have more potential to engage. Health barriers such as hearing loss, visual impairment and poor dexterity are common amongst older patient groups. These can create challenges for telemedicine, although a number of these can be overcome with simple adaptations such as headphones, patience and closed‐loop communication [16].

Inequality in digital access and utilization between socioeconomic groups is commonly cited in the literature [17]. This study supports previous findings with trends suggesting more deprived patients are less likely to have internet access, use the internet daily, desire virtual healthcare or digital monitoring, and more likely to require help with online tasks. Therefore, the progressive digitalization of healthcare risks exacerbating the current divide with the poorest and most vulnerable groups perhaps most at risk. Poor access to digital devices in lower income groups and poor digital skills contribute to the issue and while solutions are likely to be challenging and resource heavy, improvements in this area can be achieved. For example, a study in Singapore showed older adults with low socioeconomic status had a significantly higher digital literacy score following a one‐to‐one home‐based digital literacy program [18]. Zhang also suggests that if digital equity cannot be achieved from a direct approach, the investment of savings made from other more efficient digital healthcare pathways and platforms could be redirected to these underserved groups [17].

It is important to consider the perspective on telemedicine from both the patients' and healthcare professionals' points of view. This study revealed that a slim majority desire digital monitoring (57.6%) and virtual appointments to complement their face‐to‐face reviews (54.4%). For those who would prefer to continue with traditional appointments, the rationale for this was not delineated in this study but has been studied extensively elsewhere. Barriers in telemedicine include the limited scope of a medical review which lacks physical assessment [19]. Other barriers do not relate to lack of desire for a telemedicine approach, but more to resistance and uncertainty around the process, for which there may be solutions to prevent exclusion from virtual care. Complex software can be made more user‐friendly and intuitive [20], demonstration and patient/caregiver education could aid set‐up/login concerns, reassurance of secure and encrypted platforms may alleviate privacy and confidentiality anxiety [21]. Implementing digital technology in healthcare also requires engagement from the healthcare team. Although integration of telemedicine is usually viewed favorably amongst medical professionals [22], concerns include the need for duplicate appointments when virtual reviews are insufficient and the impact on the patient‐clinician relationship [23]. Outpatient appointments for patients with pulmonary hypertension include blood tests (NT‐proBNP), 6‐min walk testing (6MWT) and physical examinations. Capillary NT‐proBNP testing [24] and remote 6MWT [25, 26] have shown promise as methods to remotely obtain objective markers of pulmonary hypertension status. If these are reliable and reproducible, this could be enough to satisfy a clinician that virtual review is sufficient at that time. A hybrid approach of alternating virtual and face‐to‐face appointments may provide a compromise that takes advantage of the benefits of both.

The discrepancy between current online exercise resource users (10.5%) and potential users in our patient group (63.0%) highlights this as an area for development. In the patient focus group during the development of this questionnaire, some felt they were “stared at” in their local gym and pulmonary rehab class due to their degree of breathlessness for their age and stopped going. There was a strong desire for PH‐specific exercise resources which could cater for their degree of impairment and foster a sense of “community.” The idea of online group classes and exercise videos was well received. Future work should include the creation of a pool of online exercise content which is suitable for those with pulmonary hypertension of varying degrees of impairment.

### 3.1 Limitations

No gender data, level of education or employment data was gathered in this study. The questionnaire was only provided in English. While it could have been translated if requested, and no language‐based feedback was received, it is unknown whether any blank questionnaires returned were due to this factor which may have led to underrepresentation from non‐English‐speaking communities.

## 4 Conclusion

Most patients have the means of accessing the internet, however, access alone is not enough with a multitude of factors influencing overall digital engagement. Greater digital poverty and lower desire for virtual healthcare, digital health monitoring and online exercise resources were associated with older age and more deprived patients. Patients in general support virtual healthcare and digital health monitoring to complement face‐to‐face consultations. Two‐thirds of the PH population studied desire online exercise resources, though this is weighted towards younger patient groups. Future work should include the development of online exercise resources tailored to patients with pulmonary hypertension. The high response rate in this survey supports the use of paper, in‐person methodology for future digital inclusion research.

This study is uniquely placed in describing the current digital inclusion and patient perspective of telemedicine of the pulmonary hypertension population of Scotland. However, the authors believe the study results could have wider applicability to other patient populations. As telemedicine is progressively integrated into healthcare, systemic change to overcome barriers is vital, but the patient perspective on virtual healthcare must be kept in mind with adequate provision of traditional patient‐clinician interaction when appropriate. Consideration to this data and to digitally excluded populations should be given in all digital healthcare provision with inclusion of methods to close the digital divide.

## Author Contributions

**Jamie Ingram:** conceptualization, methodology, data curation, validation, investigation, formal analysis, visualization, writing – original draft. **Harrison Stubbs and Stephanie Lua:** conceptualization, writing – review and editing. **Melanie Brewis and Martin Johnson:** supervision, writing – review and editing. **Colin Church:** conceptualization, validation, supervision, writing – review and editing. All authors approved the final version.

## Ethics Statement

The West of Scotland Research Ethics Service were contacted and advised that this study did not require ethical review.

## Conflicts of Interest

The authors declare no conflicts of interest.

## Guarantor

Dr Colin Church is the guarantor for this study and take full responsibility for the integrity of the work.

## Data Availability

The data that support the findings of this study are available under a CC‐BY license from the University of Glasgow Enlighten: Research Data repository at DOI: 10.5525/gla.researchdata.2021.
